# From Laboratory Data to Clinical Insight The Impact of Measurement Uncertainty in Substance Screening

**DOI:** 10.1192/j.eurpsy.2026.10882

**Published:** 2026-07-31

**Authors:** A. Yıldız, Z. S. Evren

**Affiliations:** Medical Biochemistry, Başakşehir Çam and Sakura City Hospital, Istanbul, Türkiye

## Abstract

**Introduction:**

Urine drug screening is a cornerstone of addiction management, providing data to evaluate treatment adherence and detect relapse. Laboratory results inherently vary due to factors such as instrument precision, reagent and calibration stability, and matrix effects. This variability is expressed as measurement uncertainty (MU), defined as the range around a reported result within which the true value is expected to lie. As analytical conditions vary among laboratories, MU values differ between institutions. Recognizing this uncertainty helps clinicians to interpret borderline results safely and improves laboratory-clinic communication.

**Objectives:**

To estimate the MU of the urine opiate screening assay conducted in the laboratory of Başakşehir Çam and Sakura City Hospital, and to define a MU-based grey zone that supports safer and more consistent clinical interpretation.

**Methods:**

A top-down approach was applied in accordance with International Organization for Standardization (ISO) 15189 and Clinical and Laboratory Standards Institute (CLSI) EP29 guidelines. Imprecision was calculated from internal quality control data, bias from external quality assessment (LGC Group), and calibrator uncertainty was included. The combined standard uncertainty was used to calculate the expanded uncertainty (U%) with a coverage factor *k = 2.* Screening was performed with the Thermo Fisher DRI immunoassay. In total, 4555 anonymized patient results were retrospectively evaluated by applying ±U% to obtain individual uncertainty intervals.

**Results:**

The expanded MU of the opiate assay was 20.41%, calculated from internal, external, and calibrator sources. Among 4555 urine samples, 7 results (0.15%) were identified as falling within the grey zone, where the lower uncertainty limit was below and the upper limit was above the 2000 ng/mL cut-off. These findings indicate that a small number of cases near the decision threshold could potentially be misclassified if MU is not considered, highlighting its clinical relevance. A summary of analytical parameters and grey zone findings is presented in Table 1.

**Table 1.** Measurement uncertainty and grey-zone results for opiate screening Table 1. Long description.

Analytical Parameter	Opiate Screening
Cut-off (ng/ml)	2000
Expanded measurement uncertainty (U%)	20.41
Result uncertainty interval	Result ± 20.41%
Grey zone cases (n, %)	7 (0.15%)
Total samples	4555

**Conclusions:**

Applying MU to substance screening helps identify results in a clinically ambiguous zone. Integrating this MU-based grey zone into laboratory reports provides psychiatrists with a critical tool to navigate diagnostic uncertainty, thereby reducing the risk of false classification, improving the transparency of reporting, and enhancing patient safety. This approach illustrates how quality-assured laboratory data can actively guide clinical decision making in addiction care.

**Disclosure of Interest:**

None Declared

## Long descriptions

### Table 1. Long description

The table consists of two columns: Analytical Parameter and Opiate Screening.

* Cut-off (n g / m l): 2000.

* Expanded measurement uncertainty (U percent): 20.41.

* Result uncertainty interval: Result plus or minus 20.41 percent.

* Grey zone cases (n, percent): 7 (0.15 percent).

* Total samples: 4555.

Navigate back to Table 1..

